# Selenium induces a multi-targeted cell death process in addition to ROS formation

**DOI:** 10.1111/jcmm.12214

**Published:** 2014-01-08

**Authors:** Marita Wallenberg, Sougat Misra, Agata M Wasik, Cristina Marzano, Mikael Björnstedt, Valentina Gandin, Aristi P Fernandes

**Affiliations:** a Division of Pathology F46, Department of Laboratory Medicine, Karolinska Institutet, Karolinska University Hospital HuddingeStockholm, Sweden; b Department of Pharmaceutical and Pharmacological Sciences, University of PadovaPadova, Italy

**Keywords:** Selenium compounds, cell death, ER-stress, reactive oxygen species, glutathionylation

## Abstract

Selenium compounds inhibit neoplastic growth. Redox active selenium compounds are evolving as promising chemotherapeutic agents through tumour selectivity and multi-target response, which are of great benefit in preventing development of drug resistance. Generation of reactive oxygen species is implicated in selenium-mediated cytotoxic effects on cancer cells. Recent findings indicate that activation of diverse intracellular signalling leading to cell death depends on the chemical form of selenium applied and/or cell line investigated. In the present study, we aimed at deciphering different modes of cell death in a single cell line (HeLa) upon treatment with three redox active selenium compounds (selenite, selenodiglutathione and seleno-DL-cystine). Both selenite and selenodiglutathione exhibited equipotent toxicity (IC_50_ 5 μM) in these cells with striking differences in toxicity mechanisms. Morphological and molecular alterations provided evidence of necroptosis-like cell death in selenite treatment, whereas selenodiglutathione induced apoptosis-like cell death. We demonstrate that selenodiglutathione efficiently glutathionylated free protein thiols, which might explain the early differences in cytotoxic effects induced by selenite and selenodiglutathione. In contrast, seleno-DL-cystine treatment at an IC_50_ concentration of 100 μM induced morphologically two distinct different types of cell death, one with apoptosis-like phenotype, while the other was reminiscent of paraptosis-like cell death, characterized by induction of unfolded protein response, ER-stress and occurrence of large cytoplasmic vacuoles. Collectively, the current results underline the diverse cytotoxic effects and variable potential of redox active selenium compounds on the survival of HeLa cells and thereby substantiate the potential of chemical species-specific usage of selenium in the treatment of cancers.

## Introduction

Selenium is an essential trace element involved in several important intracellular processes mainly by its incorporation as selenocysteine into selenoproteins. The human genome harbours 25 selenoprotein genes. However, because of alternative splicing of RNA, the numbers of selenoprotein isoforms are higher 1. The function of selenium as an antioxidant is conferred by some of these selenoproteins that directly protect against oxidative stress. Additionally, the regeneration and activation of low molecular weight antioxidants (Q_10_, Vitamin C and E *etc*.) are mediated by the essential selenoenzyme thioredoxin reductase, and through this enzyme, selenium is a key in the defence against oxidative stress 2. Maintenance of an optimal antioxidant status is associated with efficient scavenging of free radicals and elimination of oxidants as implicated in genomic instability 3, thereby lowering the risk for cancer. In accordance, selenium compounds have been shown to prevent tumour development 4,2.

The effect of selenium is dose-dependent and chemical species-specific. The redox activity is critical as underlined by the dissatisfactory results in prevention studies where the redox inactive selenomethionine is used 5. At high levels, selenium compounds may cause extensive formation of reactive oxygen species (ROS) and induce pronounced oxidative stress 2. Although concentration dependence is one of the central characteristics of many essential nutrients, selenium is unique. Selenite is selectively toxic to tumour cells inducing cell death at concentrations that do not affect normal cells 6,7. This property indicates that selenium is a promising chemotherapeutic agent 8. The foremost mechanistic hypothesis to explain the chemotherapeutic effects of selenium is pro-oxidant activity leading to oxidation of important cellular macromolecular structures followed by induction of cell death in tumour cells 2. One of the fundamental aspects of selenium as a chemotherapeutic agent is that the therapeutic window *in vitro* and *in vivo* reside at the sub-toxic dose that is clinically achievable 9,10. Recently, we have partially decoded the mechanism of tumour selective cytotoxicity by showing the importance of extracellular thiols for uptake of selenium from selenite 11. The extracellular and intracellular thiol content are known to be elevated in many tumour types and the higher levels of thiols confer resistance against several chemotherapeutic drugs *via* thiol conjugation and detoxification 12. While protecting cancer cells from cytostatic drugs, the efficient efflux of thiols to the extracellular environment by these cells facilitates the uptake of selenide (HSe^−^) and potentiates its toxicity 11.

From a chemotherapeutic point of view, it is very important to elucidate the mode of cell death for the various selenium compounds and to explore if the differences are solely attributed to the compound used and/or to the model system. On these bases, we have studied in depth the cell death mechanisms in a single cell line (HeLa) by using three different redox active selenium compounds [selenite, selenodiglutathione (GS-Se-SG), seleno-DL-cystine], with diverse molecular structures and chemical properties. Our approach was to first investigate the morphological changes of the HeLa cells upon different selenium treatments. On this basis, we investigated the alterations in the expression of genes and proteins associated with the pathways, leading to the execution of the cell death. The choice of pathways investigated was based on the morphological characteristics of the HeLa cells treated with different selenium compounds. Finally, we have attempted to deliver the likely explanation for the activation of varied cell death modes by different selenium compounds.

## Material and methods

### Chemicals

Bovine serum albumin (BSA), sodium selenite, seleno-DL-cystine were purchased from Sigma-Aldrich (Steinheim, Germany). Necrostatin-1 (Nec-1) and neutral red dye from Sigma-Aldrich (St. Louis, MO, USA). z-VAD-fmk from Promega (Madison, WI, USA). Selenodiglutathione (GS-Se-SG) from PharmaSe (Lubbock, TX, USA). RPMI 1640 media, fetal bovine serum (FBS) (South America origin) from Gibco (Paisley, United Kingdom), and hydroethidine from Molecular Probes (Eugene, OR, USA).

### Cell culture

HeLa cells were cultured in 75 cm^2^ culture flasks (Sarstedt, Helsingborg, Sweden) in RPMI 1640 media supplemented with 10% heat-inactivated FBS at 37°C in a humidified atmosphere with 5% CO_2_. Cells were seeded at a density of 7 × 10^4^ cells/ml and incubated overnight. Prior to treatment, cells were washed once with PBS followed by addition of selenite (5 μM), GS-Se-SG (5 μM) or seleno-DL-cystine (100 μM), dissolved in RPMI 1640 media and incubated for different time-points up to 48 hrs. Culture conditions pertaining to specific experiments have been described in the pertinent sections.

### Viability measurements

Cell viability was determined by using the XTT cell proliferation kit II (Roche, Mannheim, Germany) following the manufacturer's instruction. Neutral Red uptake assay was performed under an identical culture condition as with the XTT assay with slight modification, as described by Borenfreund and Puerner 13. For the clonogenic assay, HeLa cells (1.5 × 10^5^) were seeded in 5 cm Petri dishes (Sarstedt) in 4 ml growth medium. After 24 hrs, the medium was replaced and the cells were incubated for 3 hrs in the presence of increasing concentrations of the selenium compounds. Aliquots of 200 cells were seeded in growth medium and incubated for 12 days. The colonies were stained and counted, discarding colonies with less than 50 cells. The efficiency of clonal growth (ratio between the number of colonies formed and the number of cells seeded) was calculated. The plating efficiency was about 75%.

### Quantitative PCR

Extraction of total RNA was performed by using RNeasy Plus Mini kit from Qiagen (Hilden, Germany) and with the optional on column DNase digestion, following the manufacturer protocol. RNA was quantified by using a Nanodrop® Spectrophotometer ND-1000 (Thermo Fisher Scientific, Wilmington, DE, USA). Synthesis of cDNA was performed by using an Omniscript Reverse Transcription Kit (Qiagen), by using 2 μg RNA and 0.1 μg/μl oligo(dT)_12-18_ as primer. To investigate the apoptotic pathways, pre-coated 96-wells StellARray™ (Catalog # 00188213; Lonza Bioscience, Walkersville, MD, USA) were used and performed according to the manufacturer's protocol. Real-time PCR with 20 ng of cDNA/reaction in a final volume of 20 μl was performed in a CFX96™ Real-Time System (Bio-Rad, Solna, Sweden) by using SyberGreen Supermix (Bio-Rad). Ct-values were analysed online through Global Pattern Recognition™ analysis tool, as provided by Lonza Bioscience.

### Total and oxidized intracellular glutathione measurement

Following exposure to selenite (5 μM), GS-Se-SG (5 μM) or Se-DL-cystine (100 μM) at 12 and 24 hrs, cells were washed twice with PBS, treated with 6% metaphosphoric acid and kept on ice for 15 min., followed by centrifugation. The pellet was dissolved in RIPA buffer and the protein content was determined. Aliquots of the supernatant were neutralized with Na_3_PO_4_ and assayed for oxidized glutathione following the procedure reported by Bindoli *et al*. 14.

### Selenium analysis

To study total cellular selenium accumulation, HeLa cells were exposed to selenite (5 μM), GS-Se-SG (5 μM) and Se-DL-cystine (100 μM) for 6 and 12 hrs, as described previously 15. Cellular selenium accumulation data were normalized to the protein content of the samples. To determine the protein-bound selenium, cells were treated with Se-DL-cystine (100 μM) for 3, 6, or 12 hrs. Selenium content in the protein extract was analysed as described above. To study the DNA-bound selenium, DNA was extracted and purified by using a commercial spin column quantification kit (DNeasy Blood and Tissue Kit; Qiagen). Only highly purified samples (A260/A230 ≅ 1.8 and A280/A260 ≅ 2.0) were included for analysis. The samples were completely dried and redissolved in 200 μl of Milli-Q water (18.2 MΩ) for at least 20 min. at 65°C in a shaking thermo-mixer and analysed for total selenium content.

### Detection of superoxide

The formation of superoxide was measured in HeLa cells after 5 hrs of treatment with the compounds, by staining with hydroethidine (0.1 μM), as previously described 15.

### Transmission electron microscopy

After treatment with all the selenium compounds for 3, 6 and 24 hrs, cells were fixated by addition of 2.5% glutaraldehyde in 0.1 M phosphate buffer, pH 7.4 at room temperature for 30 min. The cells were scraped off and transferred to microfuge tubes and stored in the refrigerator. After fixation, cells were rinsed in 0.1 M phosphate buffer and centrifuged. The pellets were post-fixed in 2% osmium tetroxide in 0.1 M phosphate buffer, pH 7.4 at 4°C for 2 hrs, dehydrated in ethanol followed by acetone and embedded in LX-112 (Ladd, Burlington, VT, USA). Ultrathin sections (40–50 nm) were cut by a Leica EM UC 6 (Leica, Wien, Austria). Sections were contrasted with uranyl acetate followed by lead citrate and examined under a Tecnai 12 Spirit Bio TWIN transmission electron microscope (Fei Company, Eindhoven, The Netherlands) at 100 kV. Digital images were taken by using a Veleta camera (Olympus Soft Imaging Solutions, GmbH, Münster, Germany).

### Western blot

Cell lysates were prepared by using RIPA buffer containing 1 mM PMSF, final concentration. Cell lysates (50 μg protein) were loaded into each well and separated by electrophoresis on a 12% SDS-PAGE gel (Bio-Rad). Gel separated samples were transferred to a 0.2 μm pore-sized membrane (Bio-Rad) by using a semi-dry transfer (40 mA, per membrane/gel), for 45 min. The membrane was dried in methanol for 15 min. and blocked in 5% dry milk dissolved in TBST (Tris Buffer Saline + Tween) for 2 hrs at room temperature, followed by overnight incubation with antibodies (Table 1) at 4°C. The membrane was washed three times in TBST and incubated with horseradish peroxidase-conjugated secondary antibodies for 1 hr at room temperature. The blot was developed by using enhanced chemiluminescence, from Perkin-Elmer (Boston, MA, USA). In the western blot experiments analysing ubiquitinylated proteins and glutathionylated proteins, 5 and 10 μg of protein were loaded per sample lane, respectively. In these experiments, the membrane was blocked with 1% BSA.

**Table 1:** List of antibodies used

Antibody	Company	Dilution
FGFR3	Novus Biological	1:500
OPG	Santa Cruz	1:500
BIM	Novus Biological	1:200
P62	Cell Signaling	1:20000
Caspase 2	Cell Signaling	1:1000
LC3II	Cell Signaling	1:1000
ERdj5	33	1:500
BIP	Cell Signaling	1:1000
CHOP	Cell Signaling	1:1000
PARP-1	Cell Signaling	1:1000
Caspase 3	Cell Signaling	1:1000
Ubiquitin	Enzo Life Science	1:2000
Glutathione	Virogen	1:2000
β-Actin	Sigma-Aldrich	1:1000
β-tubulin	Abcam	1:500

### Cell synchronization

To synchronize cells, cells were grown in the absence of serum for 72 hrs. Se-DL-cystine was added at different time-points after the addition of serum and subsequently treated for 24 hrs. Morphological changes were evaluated under light microscope (Nikon Eclipse TE300) and photographed with a Nikon TE-DH100w camera (Nikon Instruments Inc., Melville, NY, USA).

### Recovery and senescence staining

HeLa cells were treated with Se-DL-cystine (100 μM) for 18 hrs. Thereafter, cells were washed twice with PBS and fresh medium was added. Cells were monitored for the next 4 hrs at 37°C by using LEICA DM IRBE microscope (Deerfield, IL, USA) equipped with QIMAGING RETIGA-SRV camera (Burnaby, BC, Canada). Cells were further incubated for 14 days and fresh medium was added every 3–4th day and cell morphology was registered by light microscopy. At days 0, 7, 10, 12 and 14, cells were fixed and stained for beta-galactosidase activity by using Senescence Cells Histochemical Staining kit (Sigma-Aldrich, Germany) according to the manufacturer's instructions.

### Acridine orange staining

HeLa cells were seeded in eight-well slides with 5.0 × 10^5^ cells/well and treated for 12 hrs. Slides were washed with PBS, and stained with 10 μM acridine orange (Molecular Probes) for 20 min. at 37°C. Stained cells were examined by fluorescence microscopy (Olympus BX41 Cell F software; Olympus, Münster, Germany), with AO 606 nm blocking filter.

### ATP measurement

The ATP production was determined with the CellTiter Glo® Luminescent cell Viability Assay (Promega) and luminescence was measured on an FLx800 instrument (BioTek Instruments, Winooski, VT, USA), following the protocol provided by the manufacturer.

### Measurement of membrane potential

Depletion of the mitochondrial membrane potential (Δψ) was determined by measuring the fluorescence of cells stained with the dye tetramethylrhodamine methyl ester (TMRM, Molecular Probes). Cells were treated for 6 or 12 hrs and subsequently washed with PBS. They were harvested and incubated for 15 min. at 37°C in PBS with TMRM at a final concentration of 10 nM, freshly prepared from a 10 mM stock solution in DMSO. Fluorescence emissions from the 488 nm excited TMRM was collected from a flow cytometer (Becton Dickinson, San Jose, CA, USA) equipped with a 585/42-nm band-pass filter. The data were analysed by using FACSDiva software 5.3 (Becton Dickinson).

### Gel electrophoresis of DNA

DNA was extracted as described by Zhivotosky and Orrenius 16. Equal amount of DNA samples were loaded on a 1.8% agarose gel prepared with 1% TBE (89 mM Tris, 89 mM boric acid, 2 mM EDTA, pH 8.2) buffer. DNA was analysed by electrophoresis for 4 hrs at 50 V, in the presence of GelRed (Biotium, Hayward, CA, USA) and visualized under UV light source (FluorChemSP; Alpha Innotech, San Leandro, CA, USA).

### Analysis of selenium interaction with DNA

The interaction of selenium with DNA was monitored by agarose gel electrophoresis. Samples of supercoiled pUC19 DNA (0.5 μg/μl) were incubated in Tris buffer (50 mM Tris, 18 mM NaCl, pH 8.2) with 5 μM selenite or GS-Se-SG at 37°C for 3 hrs in the dark. The reaction was quenched by the addition of 3 μl of loading buffer (0.25% bromophenol blue and 30% glycerol). Samples of the reaction mixtures were loaded onto a 1% agarose gel in TBE buffer. The gels were subjected to electrophoresis for 4 hrs at 50 V, followed by staining with 0.5 μg/ml ethidium bromide overnight. Gel bands were visualized by using a UV transilluminator TM36 (UVP, San Gabriel, CA, USA) and photographed by using an Olympus digital camera equipped with Quantity One software (Bio-Rad, Hercules, CA, USA).

### Glutathionylation experiments

*In vitro* glutathionylation experiments were performed by incubating selenite or GS-Se-SG with reduced BSA, a thiol-rich protein. Briefly, BSA was reduced with 17-folds molar excess of DTT compared with thiols, at 37°C for 1 hr and successively desalted by using a PD-10 column (GE HealthCare, Stockholm, Sweden) equilibrated with Tris-EDTA (TE) buffer, pH 7.4. The thiol content of the desalted BSA fraction was measured by the DTNB method 17. Subsequently, selenite and GS-Se-SG were mixed with reduced BSA (molar ratio of selenium compounds to reduced thiol content of BSA = 2:1) and allowed to react for 10 min. at 37°C. These reaction mixtures were flash frozen in liquid nitrogen and stored at −20°C until analysis. To study the glutathionylation of membrane proteins, cells were treated with selenite and GS-Se-SG for 3 hrs. Following harvesting, equal numbers of cells from each treatment were briefly sonicated in TE buffer, pH 7.4 and centrifuged at 25,000 × g for 10 min. The supernatant was removed and the resulting pellet was stored at −20°C until analysis. Protein glutathionylation was analysed by western blot, as described previously, by using a GSH antibody and a GSH-conjugated BSA as control.

### Statistical analyses

All data are presented as mean ± SD (*N*). The IC_50_ values were calculated by using the equation Y = bottom + (top − bottom)/[1 + 10^((LogIC50-X)*HillSlope)^] (GraphPad Prism, Version 5, La Jolla, CA, USA). Subsequently, the dose corresponding to 50% cell death was interpolated from the same equation. Assumptions of parametric statistical tests (*i.e*., equality of normality and variance) were tested prior to data analysis. Pairwise comparison was performed with Student's *t*-test. For multiple comparison analysis, one-way anova followed by Tukey's multiple comparison test was employed. Mean values were considered significantly different at *P* < 0.05.

## Results

### Selenium-mediated cytotoxicity

**Figure 1:**
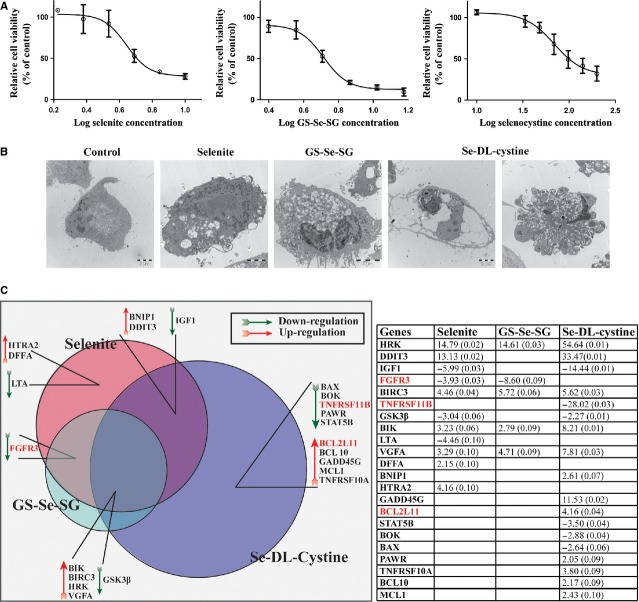
(A) Cell viability in HeLa cells was measured by XTT following treatment with increasing concentrations of selenite, GS-Se-SG and Se-DL-cystine for 24 hrs. Data are representative of three to five independent experiments. (B) Representative transmission electron microscopy micrographs of cells, illustrating the whole-cell morphology after 24 hrs of selenium treatments including untreated cells. (C) Expression profiles of mRNA levels associated with apoptosis pathway as analysed by qPCR, after treating cells with respective IC_50_ values (5 μM selenite, 5 μM GS-Se-SG and 100 μM Se-DL-cystine) for 24 hrs. Data in the Table show relative folds expression values compared with control.

In this study, HeLa cells were used as model cancer cells to dissect different cell death modes activated upon treatment with selenium compounds of different molecular structures and chemical properties: selenite, GS-Se-SG and Se-DL-cystine. On the basis of dose–response curves of cell death, we calculated the IC_50_ values for each selenium compound (5.2 μM for selenite, 5.1 μM for GS-Se-SG and 99.5 μM for seleno-DL-cystine). Similar cytotoxicity was obtained by using NR assay and clonal growth (data not shown). For convenience in dilution of stock solution, we used in the 5 μM for selenite and GS-Se-SG and 100 μM for Se-DL-cystine subsequent experiments (Fig. 1A). Morphological differences between cells treated with the different selenium compounds for 24 hrs were elucidated by transmission electron microscopy (TEM; Fig. 1B). Selenite and GS-Se-SG induced vacuolation and nuclear condensation, while Se-DL-cystine induced two different patterns of morphological changes: one population (˜50% of cells), had an apoptosis-like phenotype, while the second population had an intact cell membrane with an apparent empty cytosol.

To explore whether molecular changes followed morphological alterations, we measured the mRNA levels of 94 known apoptosis-related genes (Fig. 1C). In general, Se-DL-cystine affected more genes than selenite and GS-Se-SG, and as expected with less similarity compared with the other two selenium compounds. Uniquely, Se-DL-cystine significantly down-regulated TNFRSF11B (Osteoprotegrin, OPG; 28-fold) and up-regulated GADD45G (11.5-fold) and BCL2L11 (Bim; 4.2-fold). Selenite significantly altered the levels of LTA (4.5-fold) and up-regulated DFFA (2.2-fold) and HTRA2 (4.2-fold).

### Biochemical modification

Superoxide production, selenium uptake and thiol redox state are important features when examining the cellular responses to selenium compounds. Selenite treatment increased intracellular superoxide levels to 37.85 ± 3.38%, GS-Se-SG to 24.53 ± 3.47%, while Se-DL-cystine did not induce any significant changes (7.93 ± 2.80%) compared with the control (4.55 ± 0.49%). Both selenite and GS-Se-SG had a major impact on the intracellular ratio of reduced and oxidized glutathione (GSH/GSSG), with a fivefold increase (2.98 ± 0.65% GSSG in control and 14.23 ± 1.34% and 15.42 ± 5.54% in the selenite and GS-Se-SG treatment respectively) after 24 hrs. There was no significant difference in the selenium uptake between selenite and GS-Se-SG treatment (56.65 ± 3.32 and 48.43 ± 7.52 ng/mg protein respectively). Conversely, Se-DL-cystine treatment resulted in a much higher selenium uptake (187.76 ± 15.54 ng/mg protein), probably explained by the higher exposure concentration.

### Morphological alterations during cell death

**Figure 2:**
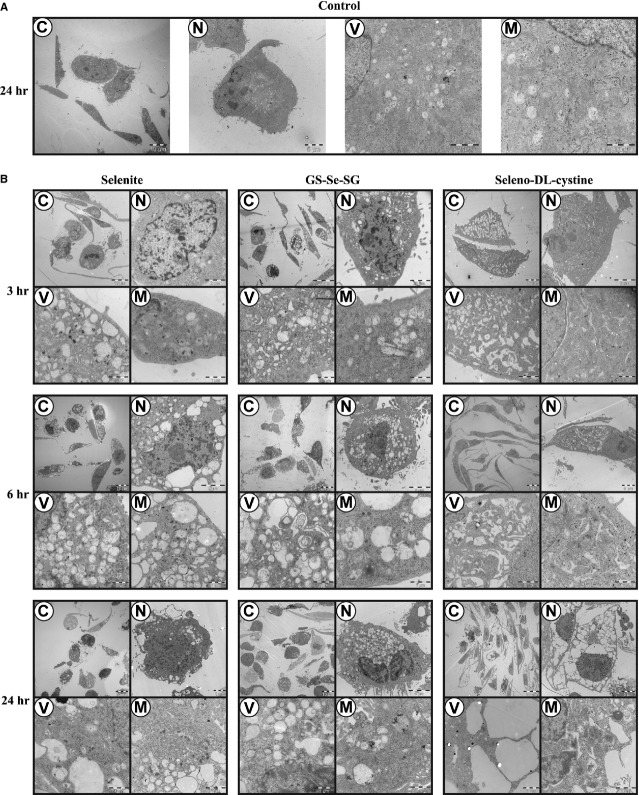
Transmission electron micrographs of cells after treatment with the three selenium compounds. (A) Untreated cells (B) cells after 3, 6 and 24 hrs of treatment with selenite (5 μM), GS-Se-SG (5 μM) and Se-DL-cystine (100 μM). C: Whole cell; N: Nucleus; V: Vacuoles; M: Mitochondria.

With the aim of better understanding the time-dependence of the effects of the selenium compounds at the cellular level, morphological changes were evaluated by TEM at different time-points (Fig. 2, Table 2).

**Table 2:** TEM evaluation of cells after treatment with selenium compounds

Time	Cell/Organelles	Compounds		
		Selenite	GS-Se-SG	Se-DL-cystine
3 hrs	Cells	+	++	N.E
	Nuclei	+	++	N.E
	Vacuoles	+	+	+
	Mitochondria	++	+	N.E
6 hrs	Cells	++	++	+
	Nuclei	++	++	N.E
	Vacuoles	++	++	++
	Mitochondria	++	++	+
24 hrs	Cells	++	++	++
	Nuclei	++	++	++
	Vacuoles	++	++	++
	Mitochondria	++	++	+

[N.E]: No effect; [+]: Mild effect; [++]: Moderate to high effect; TEM: transmission electron microscopy; FGFR3: Fibroblast growth factor receptor 3.

The concentration of selenite, GS-Se-SG and Se-DL-cystine were 5, 5 and 100 μM respectively.

Selenite and GS-Se-SG induced an early vacuolation of the cytosol, in accordance with previously reported studies with selenite 18,19. Selenite induced mitochondrial DNA condensation and mitochondrial swelling at 3 hrs of treatment, while this occurred only after 6 hrs by GS-Se-SG treatment. On the contrary, nuclear DNA condensation was more evident at 3 hrs by GS-Se-SG treatment. Selenite and GS-Se-SG, but not Se-DL-cystine treatment caused a detachment of the nuclear envelope from the cytosol. After 24 hrs’ treatment with selenite and GS-Se-SG, the cytosol was extensively filled with vacuoles, the DNA of mitochondria was condensed and nuclear DNA degraded. Se-DL-cystine triggered half of the cell population to encounter an apoptosis-like phenotype, while the second population of cells displayed a dilation of the ER, which increased over time, while the nuclei remained intact. Following 24 hrs of treatment, cells were massively vacuolated, and some of them displayed an empty cytosol.

### Protein modifications of cell death markers

**Figure 3:**
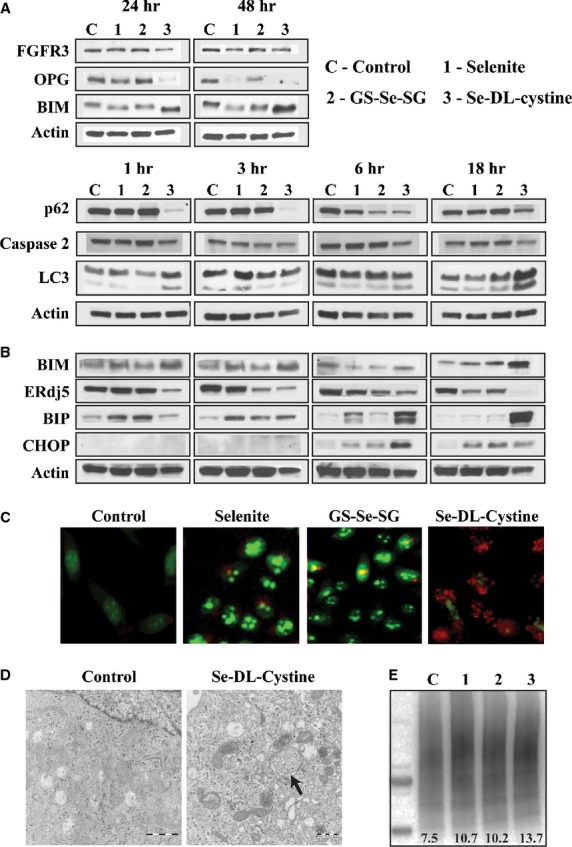
(A) Western blot analysis of apoptotic markers following 24 and 48 hrs of selenite (5 μM), GS-Se-SG (5 μM) and Se-DL-cystine (100 μM) treatment. (B) Western blot analysis of autophagic and ER stress markers, evaluated after 1, 3, 6 and 18 hrs of treatment with selenite (5 μM), GS-Se-SG (5 μM) and Se-DL-cystine (100 μM). (C) Acridine orange staining of cells after 12 hrs of treatment with selenite (5 μM), GS-Se-SG (5 μM) and Se-DL-cystine (100 μM) (magnification 40 ×). (D) High-resolution transmission electron microscopy micrographs of control and 24 hrs Se-DL-cystine-treated cells, showing the presence of autophagosome, as evident from double-layered membrane structure with inclusion bodies. (E) Western blot detection of ubiquitinylated proteins after treatments with different selenium compounds (similar doses as previous) for 6 hrs. Five microgram cell lysate was loaded into each lane. The lane intensity was analysed by using Adobe Photoshop program (version CS5). β-actin was used as a loading control (Fig. 4A and B). C: Control; 1: selenite; 2: GS-Se-SG; 3: Se-DL-cystine.

The mRNA screening (Fig. 1C) showed that different cell death related genes were activated by different selenium compounds. Selected mRNA results were thus validated at the protein level by WB (Fig. 3A). Fibroblast growth factor receptor 3 was slightly suppressed after 48 hrs incubation upon treatment with all selenium compounds. The protein level of OPG, encoded by the TNFRSF11B gene, was down-regulated by Se-DL-cystine at 24 and 48 hrs, in line with mRNA data and a similar observation was made for selenite treatment at 48 hrs. Bcl-2-like protein 11/BIM, encoded by BCL2L11 and participating in apoptosis activation, was up-regulated, in agreement with mRNA data (24 hrs), only by Se-DL-cystine (Fig. 3A and B).

To study the origin of the cytoplasmic vacuolation in the treated cells, we verified the expression levels of known biomarkers associated with autophagy and ER-stress (Fig. 3B). Only Se-DL-cystine induced LC3-II formation at 1 hr of treatment, which together with LC3-I persistently co-existed throughout the treatment (18 hrs). Protein p62 binds to ubiquitin and directs proteins designated for degradation to the autophagosomes 20 where it degrades after fusion with lysosomes. All selenium compounds caused decrease in p62 levels. Staining with acridine orange showed positivity in Se-DL-cystine treated cells, suggesting elevated lysosomal activity (Fig 3C). Cells treated with selenite and GS-Se-SG increased the chromatin condensation (nucleic acids stained with acridine orange, green colour), while a few cells stained red. Autophagosome formation was detected in cells treated with Se-DL-cystine for 24 hrs without any evidence of fusion with lysosomes (Fig. 3D). The presence of ubiquitinylated proteins was most pronounced in the cells treated with Se-DL-cystine for 6 hrs (Fig. 3E).

All selenium compounds decreased the levels of ERdj5, an ER-residing protein playing a key role in recognizing and degrading misfolded proteins. However, these changes occurred at different time-points of treatment, suggesting the activation of differential pathways leading to changes in the ER compartment. ERdj5 was down-regulated by Se-DL-cystine after 1 hr, by GS-Se-SG after 3 hrs, and by selenite after 18 hrs of treatment. At the latter time-point, ERdj5 was already abolished by Se-DL-cystine. In addition to ERdj5, we thought of interest to analyse the effect of selenium compounds on the HSP70 protein and the ER molecular chaperone Bip (GRP78), both well-known regulators of ER stress, and recognized for their interaction with other ER stress-regulating proteins, namely ERdj5 and CHOP. Increased levels of Bip were observed after 1 hr by selenite and GS-Se-SG treatments. At 3 hrs, Bip was increased by all selenium compounds, and at 6 hrs, it was cleaved by selenite and Se-DL-cystine, while GS-Se-SG treatment decreased the protein. Clear differences between the treatments were seen after 18 hrs, where the presence of Bip sustained only in cells treated with Se-DL-cystine. CHOP, which induces apoptosis as a result of ER stress, was already up-regulated at 6 hrs of incubations with all selenium compounds.

### Effects of Nec-1 on selenium cytotoxicity

**Figure 4:**
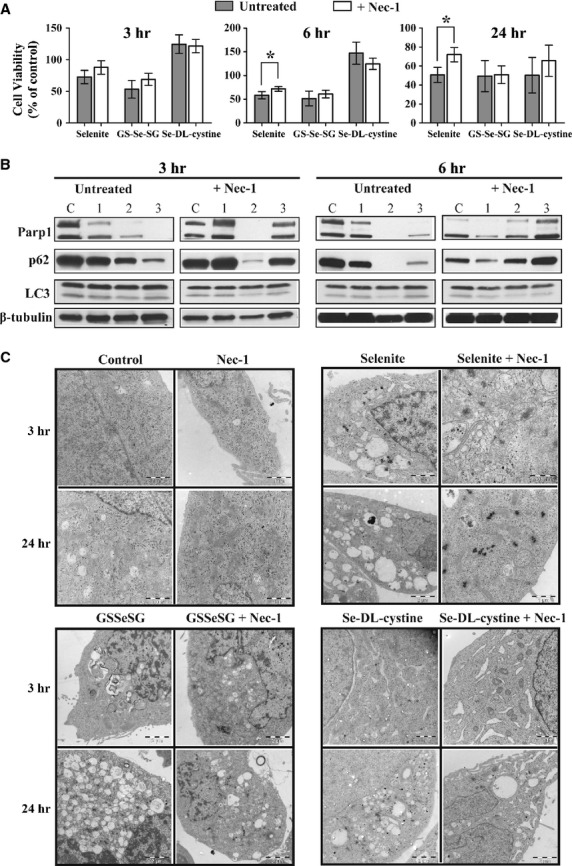
(A) Involvement of necroptosis was studied by pre-treatment with Necrostatin-1 (60 μM) for 1 hr, followed by the addition of the selenium compounds, selenite (5 μM), GS-Se-SG (5 μM) and Se-DL-cystine (100 μM). Cell viability was subsequently measured by XTT assay after 3, 6 and 24 hrs. (B) Western blot analysis of proteins associated with autophagic pathway after treatments with the selenium compounds for 3 and 6 hrs following pre-treatment with necrostatin-1. β-tubulin was used as a loading control. C: Control; 1: selenite; 2: GS-Se-SG; 3: Se-DL-cystine. (C) Transmission electron microscopy micrographs of cells treated alike as in Figure 5A and B, after 3 and 24 hrs.

The impact of a specific inhibitor of RIP 1 kinase, Nec-1 in protecting cells from necroptotic cell death was examined. Pre-treatment with Nec-1 protected cells from the cytotoxic effects of selenite (Fig. 4A). Necrostatin-1 also protected selenite-induced degradation of p62 and PARP-1 at 3 but not 6 hrs. Furthermore, Nec-1 protected Se-DL-cystine-induced p62 and PARP-1 degradation throughout the treatment. On the contrary, Nec-1 did not influence the expression pattern of PARP-1 and p62 upon GS-Se-SG treatment (Fig. 4B). The impact of Nec-1 on the morphological changes induced by the treatments was further verified by TEM. Necrostatin-1 partly protected cells from selenite-induced changes: cytosolic vacuolation was present to a lesser extent, while autophagosome-like vacuoles were abundant. To some extent, Nec-1 also protected nuclear DNA condensation, but not mitochondrial DNA condensation (Fig. 4C).

### Se-DL-cystine-induced cell death

**Figure 5:**
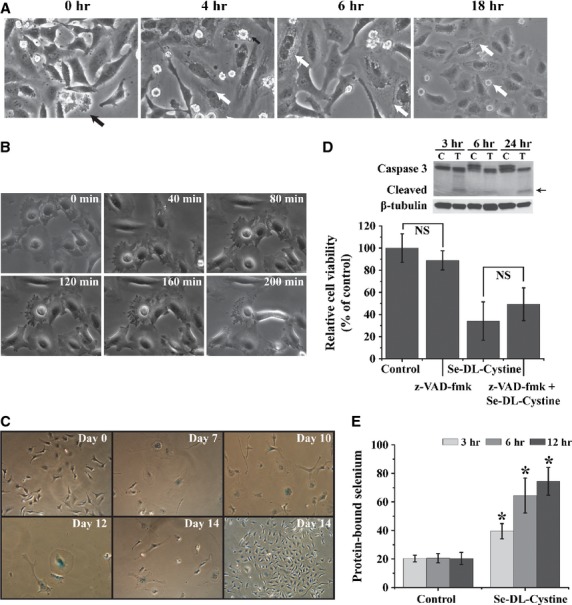
(A) Photomicrographs of cells that were serum starved for 72 hrs and subsequently treated with Se-DL-cystine (100 μM) for 24 hrs. Extensive vacuolization occurs after 6–18 hrs of treatment. (B) Time-laps photomicrographs of cells after 18 hrs of treatment with Se-DL-cystine (100 μM) and followed up for next 4 h after the removal of the treatment (Leica). (C) Senescence staining (β-galactosidase activity) of cells after the removal of Se-DL-cystine treatment that persisted for 18 hrs. (D) Experiments with the caspase inhibitor z-VAD-fmk (20 μM) was performed by co-treatment with the Se-DL-Cystine for 24 hrs. The top panel shows western blot analysis of caspase-3 and its cleaved product. ‘C’ indicates cells lysates from Se-DL-cystine-treated cells and ‘T’ indicates when z-VAD-fmk was present. The bottom panel shows cell viability as measured by trypan blue staining, either in the presence or absence of z-VAD-fmk (20 μM). (E) Protein-bound selenium in Se-DL-cystine-treated (3, 6 and 12 hrs) cells, as measured by AAS.

Since Se-DL-cystine induced two distinct types of morphological changes in treated cells, further experiments were designed to clarify this phenomenon. Cells were synchronized followed by treatment with Se-DL-cystine at different time-points after addition of FBS. Cells treated with Se-DL-cystine immediately after the addition of FBS were mostly unaffected by the treatment, with only few cells presenting apoptosis-like features. Cells treated with Se-DL-cystine at 4 hrs after addition of FBS exhibited a vacuolated morphology with an empty cytosol (Fig. 5A) even though the majority of cells had the apoptosis-like phenotype. At the later time-points, the number of vacuolated cells exceeded those with apoptosis-like morphology. The ability of cells to recover was explored by re-culturing cells in fresh medium. Following 4 hrs, the cytosolic vacuoles decreased in size and number in most cells (monitored by time laps), although some cells underwent cell death (Fig. 5B). The continuous culture of these cells for a week revealed further changes in the morphology. Many cells changed in shape and size gaining a morphology typical for senescence-like cells, and were therefore stained for beta-galactosidase activity every third day. Seven days after discarding Se-DL-cystine, cells started to appear positive for beta-galactosidase, which remained throughout the experiment (14 days). Unexpectedly, at days 13 and 14, clones of proliferating cells appeared in the culture (Fig. 5C).

The dependence of selenium-induced cancer cell death on caspase activation was verified by using the pan-caspase inhibitor z-VAD-fmk. Z-VAD-fmk significantly decreased the number of dead cells after 24 hrs of co-culture with Se-DL-cystine, indicating that the cell death mechanism, at least in part, is caspase-dependent. This was further confirmed by a cleavage of caspase 3 at 3, 6 and 24 hrs (Fig. 5D). The co-treatment of z-VAD-fmk with selenite/GS-Se-SG did not influence the number of dead cells (data not shown).

The amount of protein-bound selenium significantly increased in a time-dependent manner (Fig. 5E), thus suggesting an extensive incorporation of Se-DL-cysteine in place of cysteine, potentially leading to increased ubiquitinylation and ER stress.

### Nuclear and mitochondrial changes upon selenite and GS-Se-SG treatment

**Figure 6:**
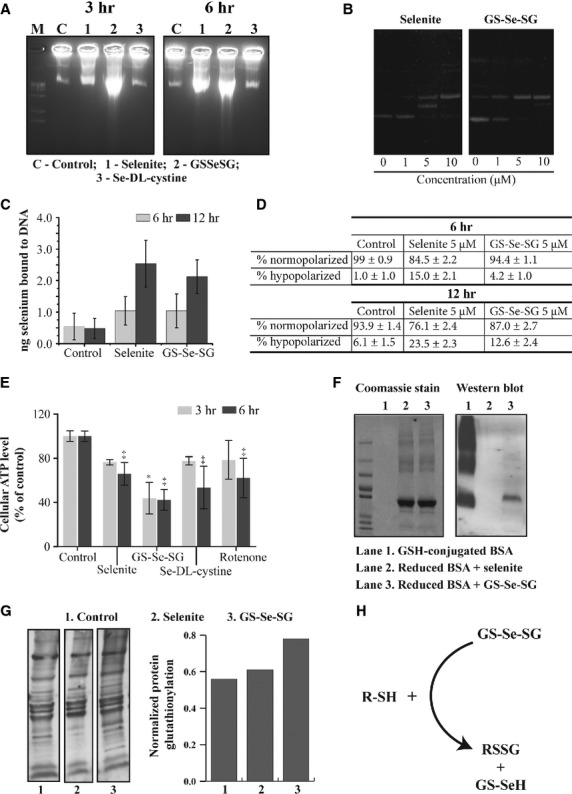
(A) DNA damage (single-strand break) after 3 and 6 hrs of treatment with selenite (5 μM), GS-Se-SG (5 μM) and Se-DL-cystine (100 μM). C: Control; 1: selenite; 2: GS-Se-SG; 3: Se-DL-cystine. DNA was isolated from equal number of cells and electrophoresed in 1.5% agarose gel. M represents molecular weight marker, size 1 kb. (B) DNA bound selenium after 6 and 12 hrs of treatment with selenite (5 μM) and GS-Se-SG (5 μM). (C) Se-bound to plasmid (pUC19) DNA after addition of increasing concentrations of selenite and GS-Se-SG. (D) Changes in the mitochondrial membrane potential as measured by flow cytometry analysis. Cells were stained with TMRM after 6 and 12 hrs of treatment with selenite (5 μM) and GS-Se-SG (5 μM). Data are representatives of at least three independent measurements. (E) Cellular ATP level after 3 and 6 hrs of treatment with selenite (5 μM), GS-Se-SG (5 μM) and Se-DL-cystine (100 μM). (F) Western blot analysis of bovine serum albumin (BSA) glutathionylation in *in vitro* experiment. 10 μg of reduced BSA was loaded in lanes 2 and 3. Note that the amount of GSH-conjugated BSA was too low to be detected by Bradford method and not visible by coomassie staining. (G) Western blot analysis (left panel) of glutathionylation of proteins derived for the pellet fractions of cells following treatment with selenite (5 μM) and GS-Se-SG (5 μM). The right panel shows quantification data as analysed by the ratio of the total lane intensity of WB to the coomassie blue staining. (H) Schematic diagram showing the mechanism of GS-Se-SG-induced protein glutathionylation.

Exposure to selenite and GS-Se-SG for 3 and 6 hrs of treatment resulted in the absence morphological evidence of nuclear DNA fragmentation. However, DNA strand breaks were detected at 3 hrs of treatment with GS-Se-SG and at 6 hrs exposure to both selenite and GS-Se-SG (Fig. 6A). *Cell-free* experiments with a DNA plasmid (pUC19) confirmed the Se-DNA interaction (Fig. 6B). The ability of selenite and GS-Se-SG to bind to DNA was also examined by atomic absorption spectroscopy (AAS; Fig. 6C). Both compounds induced selenium binding to DNA in a time-dependent manner, thus confirming their ability to interact with DNA intracellularly.

**Figure 7:**
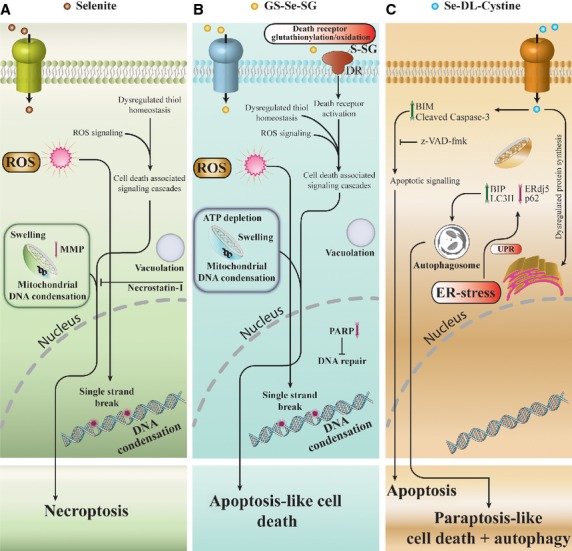
Proposed model of cell death pathways, induced by three different selenium compounds. Both selenite and GS-Se-SG exert toxic effects associated with reactive oxygen species generation, perturbation of thiols homeostasis, mitochondrial dysfunction and DNA damage. Partial inhibition of cell death by necrostatin-1 suggests the involvement of necroptosis in selenite-induced cell death. In contrast, protein glutathionylation and specific inhibition of PARP are unique to GS-Se-SG-mediated cell death. Se-DL-cystine exerts extensive ER-stress associated with unfolded protein response, followed by induction of compensatory pathways leading to protein degradation and ultimately paraptosis-like and autophagic cell death. A distinct population of cells exhibits classical morphology of apoptosis, accompanied by induction of BIM and cleavage of caspase-3. Caspase inhibitor z-VAD-fmk partially inhibits Se-DL-cystine-induced cell death. Overall, distinguished pathways of cell death are involved in selenium toxicity, depending on its chemical form.

The effect of selenite and GS-Se-SG on mitochondrial membrane potential (MMP) was determined. Selenite treatment induced a significant and time-dependent increase (15–22%) of cells with depolarized mitochondria. Similar results were achieved with GS-Se-SG, which elicited a delayed effect compared with selenite treatment, where significant changes in MMP occurred after 12 hrs (Fig. 6D). On the contrary, 3 hrs of treatment with GS-Se-SG, significantly decreased the ATP production, while selenite and Se-DL-cystine decreased ATP at the later time-point (Fig. 6E).

### Glutathionylation of proteins

Selenite and GS-Se-SG are rapidly metabolized into selenide. However, we observed clear differences between cell death mechanisms induced by selenite and GS-Se-SG. To address these differences, we hypothesized that GS-Se-SG may glutathionylate cell surface proteins and subsequently alter their function. Results from *in vitro* experiment with BSA provided evidence of protein glutathionylation specific to GS-Se-SG (Fig. 6F). The possibility of glutathionylation of the cell surface proteins was further evaluated by WB of cellular debris, containing the cell membrane. Treatment with GS-Se-SG increased the glutathionylation in the protein fraction of cellular debris by ˜25% (lane intensity analysis), compared with the control (Fig. 6G). Selenite induced only a marginal increase in protein glutathionylation. The proposed mechanism of GS-Se-SG-mediated protein glutathionylation is depicted in Figure 6H.

## Discussion

Redox active selenium compounds are potent and specific inhibitors of malignant cell growth. Several reports show that different selenium compounds have unique tumour preventive properties among the so-called antioxidants 2,21. Selenium compounds induce a complicated pattern of biochemical alterations within the cell. Hence, a single mechanism cannot account for the diverse cytotoxic effects. This complexity in the manifestation of toxic effects is of a great pharmacological advantage. It may prevent against development of drug resistance by targeting multiple pathways, unlike most of the modern cytostatic drugs affecting single or few pathways which is the basis for drug resistance. Current dogma suggests that the induction of oxidative stress is the major cytotoxicity pathway of redox active selenium compounds. Apart from it, these compounds can directly interfere with the thiol-based redox systems and redox regulation of proteins, often leading to the dysfunctions of proteins because of the oxidation of critical thiols. Reactive oxygen species-mediated cell death is very complex as it simultaneously activates several pathways, leading ultimately to the cell death 22.

The present study aimed at delineating the mechanisms involved in the cytotoxic action of three redox active selenium compounds as the redox activity is a prerequisite to cytostatic/cytotoxic effects. Our study has revealed substantial evidence that selenite and GS-Se-SG elicits diverse cytotoxic effects. Although both compounds are known to be metabolized to selenide, their mechanism of action looks remarkably different. Not only the expression patterns of mRNA and proteins were different after treatment by these compounds but also the overall cell death patterns was consistently different.

On the basis of comparison of the ultrastructural changes, we concluded that the selenium compounds initially target different intracellular compartments. Selenite induced condensation of the mitochondrial DNA earlier than the nuclear DNA, while the effect of GS-Se-SG was in the reversed order. This effect was further underlined by the single-strand breaks of the genomic DNA after 3 hrs of treatment with GS-Se-SG. Measurement of DNA-bound selenium confirmed that both selenite and GS-Se-SG lead to the increased DNA-bound selenium over time. Measurement of MMP confirmed our previous observations that the effect of selenite on mitochondria was earlier and induced more hypopolarized mitochondria, compared with GS-Se-SG treatment. Despite this, the decreased ATP production was more pronounced by GS-Se-SG and was therefore not probably caused by an altered MMP. Necroptosis described as an ordered cellular demise without the involvement of caspases is believed to originate from ROS-mediated DNA damage, overactivation of PARP-1, and eventually a decrease in ATP 23,24. A necroptosis-like cell death mode induced by the selenite treatment was supported by the partial protective effect of Nec-1, with less cytoplasmic vacuolation and increased autophagic vesicles. Necrostatin-1 also protected PARP-1 and p62 degradation at 3 hrs of selenite treatment. Moreover, less nuclear condensation and cytoplasmic vacuolation were observed, while condensation of the mitochondrial DNA persisted. No pronounced rescue effect of Nec-1 was observed against GS-Se-SG cytotoxicity.

Cell death may also be induced by activation of death receptors expressed on the cell surface. One group of these death receptors is the Fas family, known to be activated by oxidation *via* ROS formation or by glutathionylation 25,26. As both selenite and GS-Se-SG are metabolized to selenide, the prominent differences in early cytotoxic effects are most likely attributed to the extracellular cell surface modulations by GS-Se-SG. In the oxidizing extracellular environment, GS-Se-SG is a more potent oxidant, requiring only a single electron for activation compared with selenite 27. Possible products of this reaction are glutathionylated thiols and selenopersulphide derivates (GS-Se-H) that may further react with thiols and oxygen. In *in vitro* experiments, we showed that reduced thiols in albumin were efficiently glutathionylated by GS-Se-SG. This observation was further confirmed by increase in glutathionylated proteins in the membrane containing fraction of cells exposed to GS-Se-SG compared with control cells. We believe that GS-Se-SG-dependent membrane protein glutathionylation, in addition to glutathionylation of intracellular proteins, may offer a plausible explanation of the differences in toxicity between selenite and GS-Se-SG. GS-Se-SG-dependent glutathionylation of proteins thiols is a novel mechanism that merits further investigation.

The cytotoxic mechanism for Se-DL-cystine is fundamentally different compared with selenite and GS-Se-SG. Se-DL-cystine had no early effect on the nuclear DNA, but induced dilation of ER. However, Se-DL-cystine exposure leads to two morphologically distinct cell populations, one of which was characterized by classical apoptotic morphology. Morphological evaluation along with extensive modification of ER-localized proteins corroborates the findings associated with induction of ER-stress by the Se-DL-cystine treatment. An initial cleavage of LC3 at 1 hr and later on at 18 hrs indicates the possible involvement of autophagy. Changes in the expression of ER stress-regulating proteins (BIM, BIP, CHOP and ERdj5) caused by unfolded protein response (UPR) were observed after Se-DL-cystine treatment. A positive staining with acridine orange suggested an increased lysosomal activity. Although we did not detect clear evidence of autophagolysosomes, the presence of autophagosomes was observed, suggesting a clean-up mechanism, triggered by misfolded proteins 28. As the tRNA for cysteine cannot discriminate between cysteine and selenocysteine, a substantial random incorporation of selenocysteine is expected. The chemical property of selenocysteine is very different from cysteine and will therefore cause an accumulation of misfolded proteins, which will eventually lead to ER stress and UPR 29. An accumulation of protein-bound selenium and increased ubiquitinylation of proteins supported this hypothesis. HeLa cells treated with Se-DL-cystine recovered from ER stress, as evident from morphological evaluation. Some cells, however, underwent apoptosis, while other cells acquired senescence-like morphology and were positive for β-galactosidase staining. The atypical morphology of cells treated with Se-DL-cystine treatment had the features of paraptosis-like cell death, characterized by extensive cytoplasmic vacuolation 30. There is no consensus as to the molecular basis of paraptosis; however, the common feature is the dilation of ER and/or mitochondria swelling 31. Certain agents like currently studied Se-DL-cystine or celastrol 32 can co-induce more than one type of programmed cell death at the same time: apoptosis, autophagy and paraptosis and thereby increasing their therapeutic potential.

Our data demonstrate the unique multi-target response by selenium compounds on cancer cells, properties of great importance to prevent drug resistance. The cell death mode of selenite, known to mainly induce ROS formation, was predominantly necroptosis. For the first time, we show that GS-Se-SG is able to glutathionylate protein thiols, which might lead to activation of the extrinsic pathway of cell death. This can explain the distinct early response by GS-Se-SG compared with selenite. Se-DL-cystine did not induce ROS formation, but instead ER-stress, because of UPR response, putatively due to misincorporation of selenocysteine in place of cysteine. Taken together, our results clearly demonstrate the complexity in selenium-mediated cytotoxicity, valuable for future therapeutic research.
